# Optimal timing of salvage intratympanic steroids in idiopathic sudden sensorineural hearing loss

**DOI:** 10.1002/lio2.909

**Published:** 2022-09-14

**Authors:** Yongzhen Wu, Zijun Song, Yi Wang, Hui Zhao, Tongli Ren, Jianghua Jing, Na Gao, Liang Qiao, Jing Wang

**Affiliations:** ^1^ Department of Otology and Skull Base Surgery Eye Ear Nose & Throat Hospital, Fudan University Shanghai China; ^2^ National Health Commission Key Laboratory of Hearing Medicine Shanghai China; ^3^ Department of Gastroenterology Shanghai General Hospital Shanghai Jiao Tong University School of Medicine Shanghai China

**Keywords:** idiopathic sudden sensorineural hearing loss, intratympanic steroids, locally weighted regression, pure tone audiometry, salvage treatment

## Abstract

**Background:**

Salvage intratympanic steroids (ITS) works in patients with idiopathic sudden sensorineural hearing loss (ISSNHL) after failure of initial therapy, but optimal timing of administration is unknown.

**Methods:**

Two hundred and seventy patients with ISSNHL were included. Among them, 180 were treated with ITS and standard medical treatment (SMT) and the other 90 received SMT along. The hearing threshold before and after salvage treatment were compared. The relationship between the salvage starting time and hearing recovery was analyzed.

**Results:**

The hearing of ITS group improved more than that of the SMT group in all frequency bands. The effect of both strategies decreases with the delay of the starting time. ITS can improve hearing even if being administrated 5 weeks after onset while SMT failed after 3 weeks.

**Conclusion:**

Patients with profound ISSNHL can obtain extra hearing recovery from salvage ITS. The earlier salvage starts, the greater the patient benefits.

## Graphical abstract

Patients with profound idiopathic sudden sensorineural hearing loss (ISSNHL) and poor initial systemic steroids efficacy can obtain extra hearing recovery from salvage Intratympanic steroids (ITS), especially in low and medium frequencies. The earlier the salvage treatment starts, the greater the patients benefit.

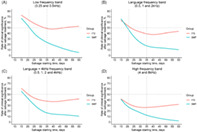

## 1 INTRODUCTION

Idiopathic sudden sensorineural hearing loss (ISSNHL) is commonly defined as a hearing loss of at least 30 dBHL in three consecutive frequencies within 3 days,^1^ characterized by a relatively high incidence (5–27 per 100,000 people per year)^2^, ^3^ and a tendency and probability for improved hearing after treatment.^4^

Systemic steroid administration has been recognized as the primary treatment for ISSNHL for over 30 years^5^ and found to increase the probability of hearing recovery by 49%–89%.^6^ However, due to the various side effects, especially in patients with diabetes, hypertension, severe gastric ulcers, and severe bone porosity, for whom the systemic steroid should be administered with caution. Therefore, there is a current increase in the use of intratympanic steroids (ITS) as an alternative or complement^7^ to systemic steroid administration, and as salvage treatment that is initiated 2 weeks after the onset of symptoms. On the other hand, the recovery of ISSNHL from initial systemic steroid treatment is not satisfactory,^8^ in these cases, salvage ITS should be applied.^9^ Many studies confirmed that ITS is an effective salvage treatment for patients with ISSNHL who have poor response or have not been exposed to conventional treatments, such as systemic glucocorticoid use. A meta‐analysis found that the hearing threshold of patients who received a salvage ITS of dexamethasone was statistically lowered.^10^ Zhou et al.^11^ reported that salvage ITS treatment is effective in patients aged >60 years, with hearing loss >70 dBHL, high‐frequency hearing loss, severe dizziness, or ISSNHL having a poor prognosis after treatment initiation more than 2 weeks from symptom onset.^4^, ^12^, ^13^, ^14^

Nevertheless, although salvage ITS has been widely used in patients with ISSNHL, no consensus has been reached on when to use it. An online survey on 1142 otolaryngologists revealed considerable differences in the treatment of ISSNHL in different countries.^15^ Thus, there is a need to explore the relationship between the salvage starting time and the efficacy of salvage ITS in the treatment of ISSNHL, defined the optimal salvage starting time, and guide the clinical practice.

Therefore, we aimed to analyze the clinical data of patients with ISSNHL complete deafness who underwent ITS salvage treatment in our hospital over the past 10 years. Moreover, we evaluated the influence of different salvage starting times of ITS salvage treatment on the changes in hearing threshold in various frequency bands of patients with ISSNHL, to find an effective strategy for the improvement of ITS salvage treatment efficacy.

## 2 MATERIALS AND METHODS

### 2.1 Ethical considerations

The study was approved by the Ethics Committee of the participating hospital. Since it is a retrospective study, we submitted an application to the committee to waive the informed consent form, and it was approved. The patients' records and information were anonymized before analysis.

### 2.2 Patients and standard treatment

We retrospectively screened the medical records of the patients with profound ISSNHL and poor efficacy after systemic steroids from January 2010 to December 2019. ISSNHL is defined as pure tone audiometry (PTA) (arithmetic average of 0.5, 1, 2, and 4 kHz air conductance threshold ≥90 dBHL).^1^ [Clinical Practice Guideline: Sudden Hearing Loss (Update)].

We included patients (1) with poor initial systemic steroid treatment efficacy, in whom hearing did not considerably improve after 2 weeks following treatment initiation, and the average hearing threshold of 0.5, 1, 2, and 4 kHz remained ≥90 dBHL; (2) who underwent salvage treatment after 2 weeks of ISSNHL onset (3) who had complete PTA results before and after salvage treatment within 1 week; and (4) no abnormality was found in MRI or CT that may lead to sudden deafness.

Finally, 270 patients were included in this study and divided into two groups based on the type of salvage treatment they received. Patients in the ITS group (*n* = 180) received salvage ITS (dexamethasone 5 mg/ml, 0.5 ml tympanic injection, 3 times a week, at least 2 weeks). If the hearing improvement in any frequency band exceeded 10 dBHL, the treatment duration was prolonged by 1 week; however, if the hearing did not improve significantly, the ITS treatment was stopped. The other 90 patients who rejected the salvage ITS were taken as controls [standard medical treatment (SMT) group, *n* = 90]. All of the 270 patients had received SMT including hyperbaric oxygen therapy, gingko biloba extract, and mecobalamin. In HBO therapy protocol, all patients inhaled 99%–100% pure oxygen when they were exposed to 2.2 atmosphere absolute in a specially designed chamber, with treatment 5 times a week, 20 times in total.

Each group was further divided into three subgroups based on the salvage starting time (the time from disease onset to the initial of salvage, <3 weeks, 3–5 weeks, and >5 weeks) to clarify its effect on the efficacy of salvage treatment.

### 2.3 Assessment of hearing improvement

Air conduction hearing threshold in PTA at 0.25, 0.5, 1, 2, 4, and 8 kHz were measured before treatment and once a week during the treatment period. The average hearing threshold of following four frequency bands was calculated: low‐frequency band (0.25 and 0.5 kHz), language frequency band (0.5, 1, and 2 kHz), language frequency + 4 kHz frequency band (0.5, 1, 2, and 4 kHz), and high‐frequency band (4 and 8 kHz). The hearing threshold before and after salvage treatment and the hearing improvement was compared between the two groups of patients and between the subgroups of different salvage starting time. The hearing threshold of one frequency band decreased by 15 dBHL or more after salvage treatment was considered to be clinical significance in the corresponding frequency band. The clinical significance rate was calculated and compared between ITS and SMT groups and their subgroup as well.

### 2.4 Statistical analysis

Continuous variables are presented as mean ± standard deviation (SD), and categorical variables are presented as frequency (percentage). To clarify how different salvage starting time affect hearing threshold improvement of the salvage treatment, box plots and paired t‐tests were used to compare the changes of the hearing threshold between the ITS and SMT groups under the same salvage starting time (<3 weeks, 3–5 weeks, and >5 weeks), respectively. The dynamic relationship between the patients' clinical significance rate in each frequency band and the salvage starting time was intuitively plotted by Locally Weighted Regression (LOESS). In all statistical tests, a two‐side *p* value <.05 was considered to be statistically significant. Statistical analyses were performed using R software version 4.0.0 (https://www.r project.org/).

## 3 RESULTS

### 3.1 Clinical characteristics before salvage treatment

A total of 180 patients were included in the ITS group, and 90 patients were included in the SMT group. There were no significant differences in age (44.7 ± 15.2 vs. 43.3 ± 15.8, *p* = 0.821), sex (male accounting for 35.6% and 33.3%, *p* = .478), salvage starting time (23.9 ± 9.0 vs. 25.5 ± 9.6 days, *p* = .201) and the hearing threshold of each frequency and frequency band between the ITS and SMT groups (Table 1).

**TABLE 1:** Characteristics of patients from ITS and SMT groups before salvage treatment

Variable	ITS group	SMT group	*P* value
	*N* = 180	*N* = 90	
Demographics			
Age, years	44.7 ± 15.2	43.3 ± 15.8	.821
Male, *n* (%)	64 (35.6)	30 (33.3)	.478
Salvage starting time,^a^ day	23.9 ± 9.0	25.5 ± 9.6	.201
Hearing threshold, dBHL			
0.25 kHz	95.9 ± 20.6	96.8 ± 19.5	.726
0.5 kHz	99.2 ± 14.5	99.8 ± 13.3	.749
1 kHz	104.4 ± 13.1	103.3 ± 12.0	.510
2 kHz	107.1 ± 13.1	105.5 ± 13.0	.359
4 kHz	109.4 ± 13.2	110.5 ± 12.0	.524
8 kHz	112.9 ± 13.7	113.2 ± 12.4	.834
Average hearing threshold,^b^ dBHL			
Low‐frequency band	97.6 ± 16.8	98.3 ± 15.5	.723
Language frequency band	103.6 ± 12.2	102.9 ± 10.8	.652
Language frequency +4 kHz band	105.0 ± 11.6	104.8 ± 10.1	.862
High‐frequency band	109.8 ± 11.7	109.7 ± 10.9	.975

*Note*: Data are expressed as mean ± standard deviation or *n* (%).

Abbreviations: ITS, intratympanic steroid; SMT, standard medical treatment.

^a^ Salvage starting time means the days from disease onset to the initiati of salvonage treatment.

^b^ Average hearing threshold including low frequency band (0.25 and 0.5 kHz), language frequency band (0.5, 1, and 2 kHz), language frequency +4 kHz frequency band (0.5, 1, 2, and 4 kHz), and high frequency band (4 and 8 kHz).

We further compared the characteristics of patients receiving ITS and SMT in the subgroups of different salvage starting times respectively, no statistical difference was observed neither (Table 2). In a word, the demographic characteristics and audiologic features before salvage treatment of the two groups of patients were similar.

**TABLE 2:** Characteristics of patients from ITS and SMT group before salvage treatment (by subgroups of different salvage starting time)

Subgroups	Salvage starting time <3 weeks			Salvage starting time within 3–5 weeks			Salvage starting time >5 weeks		
Variable	ITS group	SMT group	*P* value	ITS group	SMT group	*P* value	ITS group	SMT group	*P* value
	*N* = 82	*N* = 35		*N* = 74	*N* = 39		*N* = 24	*N* = 16	
Demographics									
Age, years	45.6 ± 15.3	40.9 ± 17.0	.145	44.2 ± 14.8	45.3 ± 15.8	.700	43.2 ± 16.3	43.4 ± 13.2	.963
Male	32 (39.0)	13 (37.1)	1.000	24 (32.4)	11 (28.2)	.804	8 (33.3)	6 (37.5)	1.000
Salvage starting time, day^a^	17.3 ± 1.7	17.3 ± 1.8	.920	25.3 ± 3.5	26.1 ± 4.0	.270	42.6 ± 6.9	41.9 ± 7.1	.762
Hearing threshold, dBHL									
0.25 kHz	99.2 ± 19.7	101.6 ± 18.5	.547	93.2 ± 21.8	95.5 ± 20.1	.590	92.9 ± 18.8	89.7 ± 18.5	.595
0.5 kHz	101.7 ± 13.5	103.6 ± 13.1	.492	97.2 ± 15.2	97.3 ± 13.6	.960	97.3 ± 15.2	97.8 ± 11.7	.908
1 kHz	105.3 ± 13.0	104.1 ± 12.1	.652	103.4 ± 13.0	103.6 ± 11.9	.933	104.2 ± 13.9	100.6 ± 12.4	.415
2 kHz	108.0 ± 12.5	107.6 ± 12.3	.849	106.7 ± 13.2	104.4 ± 13.5	.378	104.8 ± 15.1	103.8 ± 13.8	.826
4 kHz	111.2 ± 11.8	111.3 ± 11.8	.958	108.3 ± 14.0	109.4 ± 11.9	.691	107.1 ± 15.0	111.6 ± 13.1	.338
8 kHz	114.0 ± 12.6	113.4 ± 12.3	.833	113.0 ± 13.0	112.8 ± 13.2	.953	108.8 ± 18.6	113.8 ± 11.5	.344
Average hearing threshold,^b^ dBHL									
Low frequency band	100.5 ± 15.9	102.6 ± 15.0	.504	95.2 ± 17.9	96.4 ± 15.8	.723	95.1 ± 15.4	93.8 ± 14.5	.782
Language frequency band	105.0 ± 11.8	105.1 ± 10.8	.974	102.4 ± 12.2	101.8 ± 11.0	.779	102.1 ± 13.3	100.7 ± 10.2	.732
Language frequency +4 kHz band	106.6 ± 11.1	106.6 ± 10.1	.968	103.9 ± 11.9	103.7 ± 10.1	.918	103.3 ± 12.6	103.4 ± 10.3	.978
High frequency band	111.1 ± 10.7	110.8 ± 10.4	.891	109.3 ± 11.7	108.8 ± 11.2	.835	106.9 ± 14.5	109.7 ± 11.4	.518

*Note*: Data are expressed as mean ± standard deviation or *n* (%).

Abbreviations: ITS, intratympanic steroid; SMT, standard medical treatment.

^a^ Salvage starting time means the days from disease onset to the initial of salvage treatment.

^b^ Average hearing threshold including low frequency band (0.25 and 0.5 kHz), language frequency band (0.5, 1, and 2 kHz), language frequency +4 kHz frequency band (0.5, 1, 2, and 4 kHz), and high‐frequency band (4 and 8 kHz).

### 3.2 Clinical outcome after salvage treatment

After salvage treatment, the average hearing threshold of each frequency band in the ITS group was significantly lower than that of the SMT group (Table 3). By comparing the change of the hearing threshold between the two groups after salvage treatment, we found more decrease of hearing threshold in ITS group than that in the SMT group, wherever in the low (25.1 ± 21.4 vs. 10.5 ± 19.0, *p* < .001), language (17.0 ± 16.6 vs. 7.7 ± 14.8, *p* < .001), language +4 kHz (15.8 ± 15.9 vs. 7.2 ± 13.8, *p* < .001), and high‐frequency bands (11.4 ± 15.9 vs. 4.3 ± 12.8, *p* < .001).

**TABLE 3:** The pure tone audiometry results of patients from ITS and SMT groups patients after salvage treatment

	ITS group *N* = 180	SMT group *N* = 90	*P* value
Average hearing threshold^a^ after salvage treatment, dBHL			
Low‐frequency band	72.5 ± 22.3	87.8 ± 20.0	<.001
Language frequency band	86.6 ± 17.4	95.2 ± 16.2	<.001
Language frequency +4 kHz band	89.2 ± 17.2	97.6 ± 15.3	<.001
High‐frequency band	98.4 ± 19.1	105.4 ± 14.8	.003
Change of average hearing threshold^b^ after salvage treatment, dBHL			
Low‐frequency band	25.1 ± 21.4	10.5 ± 19.0	<.001
Language frequency band	17.0 ± 16.6	7.7 ± 14.8	<.001
Language frequency +4 kHz band	15.8 ± 15.9	7.2 ± 13.8	<.001
High‐frequency band	11.4 ± 15.9	4.3 ± 12.8	<.001
Clinical significance,^c^ *n* (%)			
Low‐frequency band	112 (62.2)	38 (42.2)	.003
Language frequency band	93 (51.7)	34 (37.8)	.043
Language frequency +4 kHz band	88 (48.9)	30 (33.3)	.022
High‐frequency band	60 (33.3)	20 (22.2)	.081

Abbreviations: ITS, intratympanic steroid; SMT, standard medical treatment.

^a^ Average hearing threshold including low‐frequency band (125, 250, and 500 Hz), language frequency band (500 Hz, 1 kHz, and 2 kHz), language frequency +4 kHz band (500 Hz, 1 kHz, 2 kHz, and 4 kHz), and high‐frequency band (4 and 8 kHz).

^b^ The decrease of average hearing threshold after salvage treatment.

^c^ Clinically significance is defined as the average hearing threshold of the corresponding frequency band decreased of 15 dBHL or more after salvage treatment.

Clinically significance is defined as the average hearing threshold of the corresponding frequency band decreased by 15 dBHL or more after salvage treatment. The number and rate of patients got clinical significance in the four frequency bands after treatment in the two groups are shown respectively in Table 3. The rate of clinical significance of ITS group was higher than that of SMT group in the low (62.2% vs. 42.2%, *p* = .003), language (51.7% vs. 37.8, *p* = .043), and language frequency +4 kHz band (48.9% vs. 33.1%, *p* = .022), but was not statistically different in high‐frequency band (33.3% vs. 22.2%, *p* = .083).

In conclusion, the hearing of the patients in both of the two groups got improved after the salvage treatment, but the efficacy of ITS was significantly better. Moreover, it could be found that the higher the frequency, the less improvement of the hearing threshold.

### 3.3 Role of salvage starting time

**FIGURE 1:**
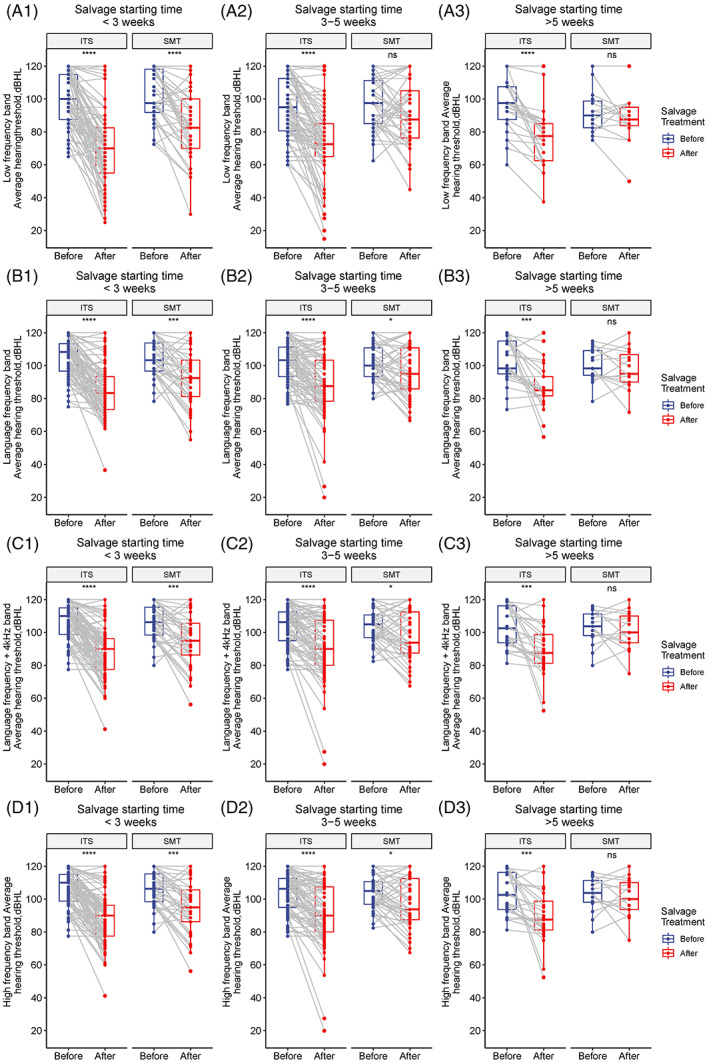
Comparison of the hearing threshold change in each frequency band before and after the salvage treatment in the intratympanic steroid (ITS) and standard medicine treatment (SMT) groups under the same salvage starting time. (A) The comparison in the low‐frequency band (0.25 and 0.5 kHz) between the ITS and SMT groups. (A‐1, A‐2, A‐3) The comparison among patients started salvage treatment <3, 3–5, and >5 weeks after disease onset respectively, the same below); (B) the comparison in the language frequency band (0.5, 1, and 2 kHz); (C) the comparison of the hearing threshold in the language frequency +4 kHz frequency band (0.5, 1, 2, and 4 kHz); (D) the comparison of the hearing threshold in the high‐frequency band (4 and 8 kHz). *p < .05, **p < .01, ***p < .001, ****p < .0001. ITS, intratympanic steroid; SMT, standard medical treatment

Figure 1 shows the effect of different salvage starting time on the hearing threshold decrease in each frequency band after salvage ITS and SMT respectively. In the low‐frequency band, whenever the salvage treatment started, the hearing threshold of the ITS group after treatment was significantly lower than that before the treatment (Figure 1A1–A3, *p* < .001 for all), while the hearing threshold of the SMT group decreased significantly only when the salvage starting time was <3 weeks (Figure 1A1–A3). In the language (Figure 1B1–B3), language +4 kHz (Figure 1C1–C3), and high‐frequency band (Figure 1D1–D3), the hearing threshold of the ITS group also decreased significantly after salvage treatment (*p* < .001 for any subgroup of different salvage starting time). As a control, though when the salvage starting time was <3 weeks or 3–5 weeks, SMT could significantly reduce patients' hearing threshold of the corresponding frequency bands; but when the salvage starting time was >5 weeks, SMT would not improve the patients' hearing any more.

**FIGURE 2:**
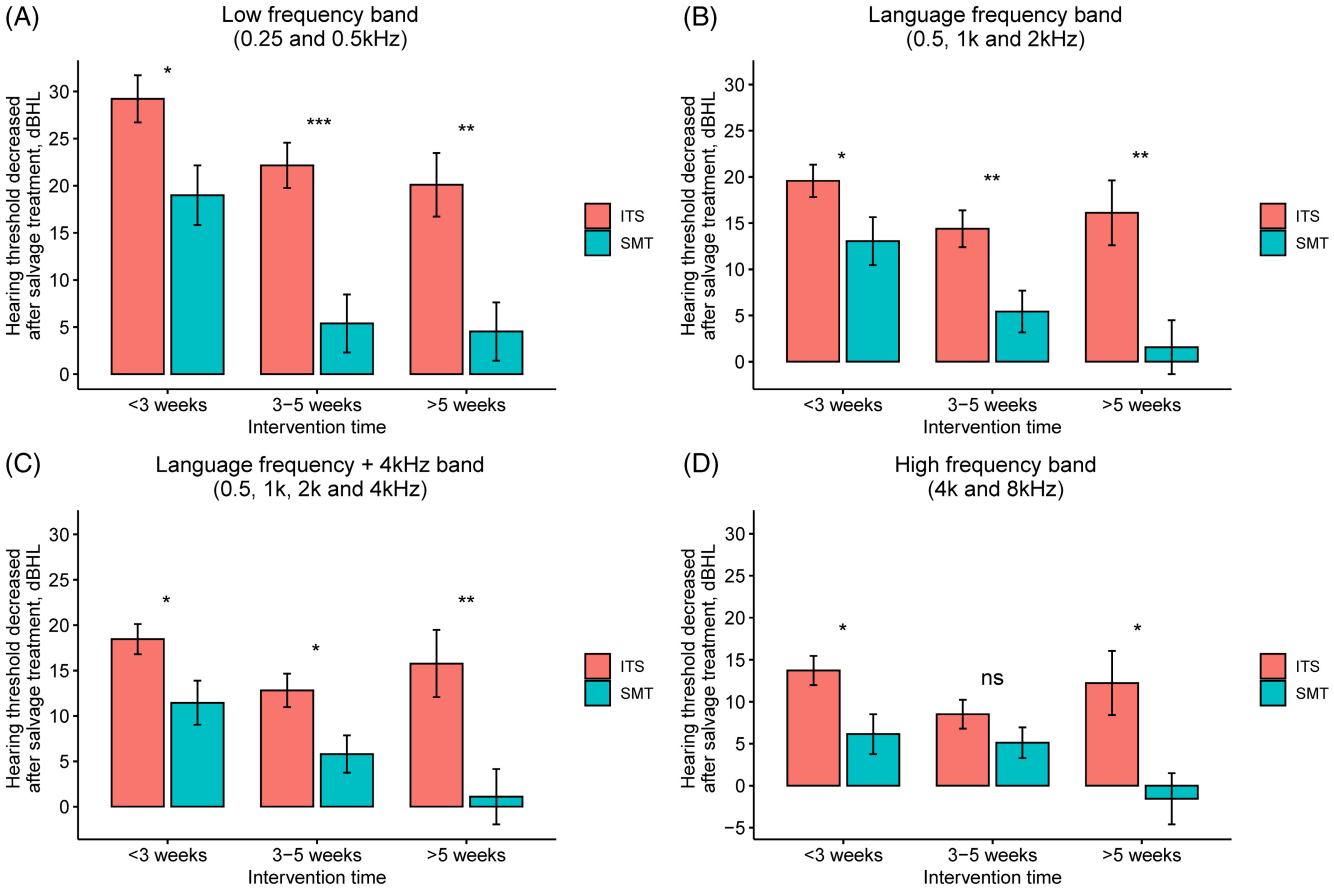
Comparison of the hearing threshold decrease in each frequency band after the salvage treatment in the intratympanic steroid (ITS) and standard medicine treatment (SMT) groups under the same salvage starting time; (A) the comparison in the low‐frequency band (0.25 and 0.5 kHz) between the ITS and SMT groups; (B) the comparison in the language frequency band (0.5, 1, and 2 kHz); (C) the comparison of the hearing threshold in the language frequency +4 kHz frequency band (0.5, 1, 2, and 4 kHz); (D) the comparison of the hearing threshold in the high‐frequency band (4 and 8 kHz). *p < .05, **p < .01, ***p < .001, ****p < .0001. ITS, intratympanic steroid; SMT, standard medical treatment.

Figure 2 shows the comparison of hearing threshold decrease between the ITS and SMT groups by different salvage starting time. In the low‐frequency band (Figure 2A), the hearing threshold decrease after salvage treatment of the ITS group was great than the SMT group, regardless of the salvage starting time (ITS vs. SMT <3 weeks: 29.2 ± 22.7 dBHL vs. 19.0 ± 18.7, *p* = .021; 3–5 weeks: 22.2 ± 20.7 dBHL vs. 5.4 ± 19.2, *p* < .001; <5 weeks: 20.1 ± 16.5 vs. 4.5 ± 12.4, *p* < .001). In the language (Figure 2B) and language +4 kHz frequency bands (Figure 2C), the hearing threshold decrease of the ITS was statistically more than that of the SMT group in any subgroup of salvage starting time as well. In the high‐frequency band, the hearing threshold decrease of the ITS group was statistically greater than the SMT group in the <3 weeks (13.7 ± 15.8 dBHL vs. 6.1 ± 14.0, *p* = .016) and >5 weeks (12.2 ± 18.7 vs. −1.6 ± 12.2, *p* = .013) subgroups, but not in the 3–5 weeks subgroup (8.5 ± 14.8 vs. 5.1 ± 11.4, *p* = .216).

**FIGURE 3:**
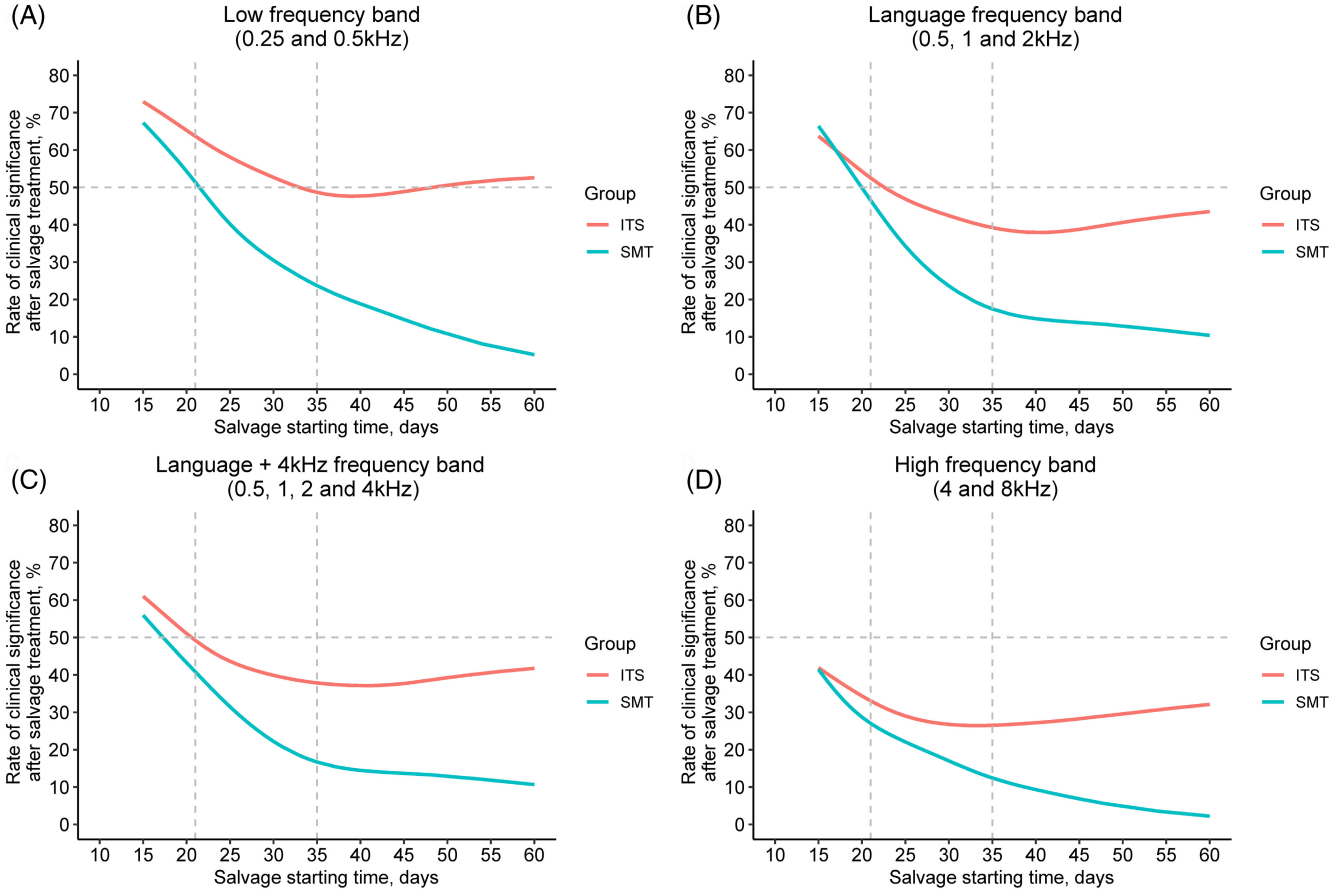
The quantitative relationship between the salvage starting time and clinical significance rate of the intratympanic steroid (ITS) and standard medicine treatment (SMT) groups. (A) the comparison in the low‐frequency band (0.25 and 0.5 kHz) between the ITS and SMT groups; (B) the comparison in the language frequency band (0.5, 1, and 2 kHz); (C) the comparison of the hearing threshold in the language frequency +4 kHz frequency band (0.5, 1, 2, and 4 kHz); (D) the comparison of the hearing threshold in the high‐frequency band (4 and 8 kHz). The horizontal line represents the clinical significance rate of 50%, and the left and right vertical lines represent the salvage starting time of 3 and 5 weeks, respectively. The clinical significance rate of the ITS and SMT groups were relatively close if the salvage treatment implemented early (<3 weeks). However, as the start of salvage delays, the clinical significance rate of the SMT group slumps much faster than that of the ITS group in all frequency bands, and their difference in the clinical significance rate becomes wider. ITS, intratympanic steroid; SMT, standard medical treatment

Finally, we used the LOESS to intuitively present the dynamic relationship between the salvage starting time and the clinical significance rate of the two groups (Figure 3). We found that if the salvage treatment was implemented early (<3 weeks), the clinical significance rate of the ITS and SMT groups were relatively close. However, as the implementation of salvage delays, the clinical significance rate of the SMT group slumps in all frequency bands, whereas that of the ITS group shows a much more gradually decline. The difference in the clinical significance rate becomes wider with the delay of salvage. We take the hearing threshold in the low‐frequency band (Figure 3A) as an example, to reach a 50% clinical significance rate, SMT has to be implemented within 3 weeks after disease onset; whereas it is not too late for ITS to be implemented even after 5 weeks. Similarly, for the change in hearing threshold after treatment in the language frequency (Figure 3B) and language frequency +4 kHz bands (Figure 3C), ITS also provides ISSNHL patients with a larger effective time window than SMT. However, neither of the two achieve a 50% hearing threshold clinical significance rate in the high‐frequency band (Figure 3D). Notwithstanding, the ITS group would maintain a clinical significance rate over 30% over 5 weeks after disease evolution, whereas the SMT group was less than 10%.

In summary, we can draw some conclusions. First of all, ITS, as a salvage treatment for ISSNHL, can effectively reduce the hearing threshold in all frequency bands, better than SMT both statistically and clinically. Secondly, the earlier the implementation, the better the efficacy. The time window of SMT is only 3 weeks, whose curative effect drops rapidly after 3 weeks and becomes invalid for more than 5 weeks. In contrast, although the curative effect of ITS also decreases with the delay of the intervention, it can still significantly reduce patients' hearing threshold even after more than 5 weeks. Finally, the higher the frequency, the worse the efficacy. The improvement of high‐frequency hearing in both groups was unsatisfactory.

## 4 DISCUSSION

We analyzed 270 patients with profound ISSNHL and poor response to initial systemic steroids, compared with the curative effect between SMT combined with ITS (*n* = 180) and SMT alone (*n* = 90) as salvage treatment. Moreover, via detailed data and intuitive plots, the influence of the salvage starting time on the curative effect was firstly elaborated. In this study, we demonstrated that SMT combined with ITS is the better salvage strategy than SMT alone in hearing recovery for profound ISSNHL, especially for patients whose salvage was implemented over 3 weeks after disease onset. The lower the frequency, the earlier the implementation, the better the curative effect is the common feature of the two salvage strategies.

The complete hearing recovery rate of ISSNHL is low. According to previous reports, the hearing recovery rate of patients with first‐time ISSNHL is only 5%–30%.^2^, ^8^, ^16^ Improving the curability of ISSNHL has always been a focus of attention. Previous studies have shown that^10^, ^17^ patients treated with salvage ITS have a greater improvement in pure tone hearing threshold. Therefore, salvage ITS may be a promising method for patients with ISSNHL having poor previous treatment efficacy. However, few studies has evaluated the ITS efficacy at different frequencies and the effect of salvage starting time on ITS efficacy. As a result, there is still no clear consensus on how to maximize the therapeutic advantages of salvage ITS. Our findings filled these knowledge gaps by revealing some important characteristics of ITS salvage treatment.

We found that patients who received ITS had a statistically more decrease of the hearing threshold in all frequency bands than those who received SMT alone, implying salvage ITS exerts further improvements on the basis of conventional vasodilators and neurotrophic drugs. Secondly, as the frequency increases, the curative effect of ITS becomes worse, manifesting as the attenuation in hearing threshold improvements and a gradual decrease in clinically significant rate. However, we also observed these patterns in the SMT group. This is probably because the low‐frequency auditory hair cells have better tolerance to hypoxia and other damage sources, whereas the high‐frequency auditory hair cells are vulnerable to damage and apoptosis.^18^, ^19^ Considering that the average age of the patients included in this study was close to 45 years old, we infer that before the onset of sudden deafness, the age‐related hearing impairment in the patients' high‐frequency band had already existed, and the room for salvage treatment to improve is not as much as that of the lower frequency bands.

The curative effect of both ITS and SMT declines with the delay of the implementation, so early salvage treatment implementation should be recommended. One of our important findings is that when the salvage starting time is >5 weeks, patients with ISSNHL will no longer benefit from SMT alone, only combining ITS can reduce their hearing threshold. Thus, ITS not only has an improved clinical efficacy, but also a better strategy in a delayed salvage scenario. On the other hand, though the hearing threshold improvement of ITS group was statistically better than that of SMT group in all frequency bands, as a noninvasive strategy for early salvage, SMT alone still has value. If receiving SMT alone within 3 weeks, patients can also obtain relatively satisfactory hearing improvement, which can be used for ISSNHL patients who are unable or unwilling to receive ITS.

We encountered several limitations in this study: first, as a retrospective single‐center analysis, it cannot be denied that there may be biases in the presented results. We adopted detailed statistical analysis and subgroup comparisons to minimize the potential bias. Second, we only included patients with profound ISSNHL, and did not include patients with hearing loss only in specific frequencies or mild ISSNHL. However, the hearing threshold of all frequencies of patients included in this study were close before salvage treatment (Table 1 and Table 2), which makes it possible to show the difference of ITS efficacy between frequency bands. Thirdly, all of the patients included in the ITS group received 2 or 3 weeks of fixed‐dose ITS treatment, and whether the dose and duration of ITS will influence the curative effect is unknown. The consistent ITS treatment plan avoided the bias that may be caused by different doses and durations. We plan to conduct a randomized controlled trials to explore the optimal dose and duration of ITS salvage treatment.

## 5 CONCLUSIONS

This study verified the role of ITS in the salvage treatment of patients with profound ISSNHL and poor response to initial systemic steroids. Compared with vasodilator and neurotrophic drugs alone, patients can obtain extra hearing recovery from salvage ITS, especially in low and medium frequencies. The earlier salvage treatment starts, the greater the patient benefit. Salvage treatment should be started within 3 weeks; if more than 5 weeks, combining ITS is strongly recommended. As non‐invasive treatment, vasodilator and neurotrophic drugs can be used for the patients who are unable or unwilling to receive ITS, but should be within 3 weeks after disease onset.

## AUTHOR CONTRIBUTIONS

Yongzhen Wu, Zijun Song, Yi Wang and Hui Zhao contributed equally and shared the first authorship. Yongzhen Wu, Yi Wang, Tongli Ren, Jianghua Jing, and Na Gao collected the data. Zijun Song and Liang Qiao contributed to the statistical analysis. Zijun Song drafted the manuscript. Jing Wang and Yongcheng Wu contributed to the critical revision of the manuscript for important intellectual content and approved the final version of the manuscript. All authors have read and approved the final manuscript. Zijun Song and Liang Qiao provided statistical support.

## FUNDING INFORMATION

This study was supported by the General Program of National Natural Science Foundation of China (NSFC 81870724), the Innovation Project of Shanghai Municipal Science and Technology Commission (19441900400), and the Shanghai Municipal Health Commission of China (201740018).

## CONFLICT OF INTEREST

The authors have no conflicts of interest to disclose.
